# Dual-Action Niclosamide–Polysaccharide Nasal Spray for the Early Therapeutic Intervention of Respiratory Viral Infections

**DOI:** 10.3390/ijms27125420

**Published:** 2026-06-16

**Authors:** Jongseo Choi, Dongjin Lee, Yujeong Na, Byeongyong Kim, Sangeun Cho, Kyungmin Lee, Kyeunghwa Chun, Gwanyoung Kim, Seong Kug Eo, Sokho Kim

**Affiliations:** 1 Daewoong Pharmaceutical Co., Ltd., 72 Dugye-ro, Pogok-eup, Cheoin-gu, Yongin-si 17028, Gyeonggi-do, Republic of Korea; 2210302@daewoong.co.kr (J.C.); leedj@daewoong.co.kr (D.L.); 2210494@daewoong.co.kr (Y.N.); 2210565@daewoong.co.kr (B.K.); sangeuncho@daewoong.co.kr (S.C.); 1471km@daewoong.co.kr (K.L.); khcheon086@daewoong.co.kr (K.C.); pharmrich@daewoong.co.kr (G.K.); 2 College of Veterinary Medicine, Biosafety Research Institute and Core Facility Center for Zoonosis Research, Jeonbuk National University, 79 Gobong-ro, Iksan 54596, Jeollabuk-do, Republic of Korea; vetvirus@jbnu.ac.kr; 3 Department of Biohealth, Kunsan National University, 558 Daehak-ro, Gunsan 54150, Jeollabuk-do, Republic of Korea

**Keywords:** influenza, xanthan gum, niclosamide, nasal spray, SKP2

## Abstract

Extensive efforts have been undertaken by numerous researchers to control respiratory viruses across the domains of diagnosis, prevention, and treatment. In this study, we developed a niclosamide–polysaccharide nasal spray (NPNS) formulation based on xanthan gum (XG), a naturally derived polysaccharide, and niclosamide, a conventional anthelmintic agent. We then evaluated its therapeutic efficacy following intranasal administration under influenza virus-infected conditions. NPNS was assessed for cytotoxicity under Good Laboratory Practice (GLP) conditions in accordance with ISO 10993-5, and no cytotoxic effects were observed. In influenza virus-infected human nasal epithelial cells (HNEc), NPNS treatment resulted in at least 92.5% suppression of viral gene expression. Furthermore, NPNS demonstrated significantly greater antiviral activity compared to Placebo 1 and Placebo 2, which were formulated by excluding niclosamide and XG, respectively. Owing to the physicochemical properties conferred by XG, NPNS exhibited prolonged retention on the nasal mucosa in a mouse model. Consistently, NPNS showed potent antiviral efficacy in influenza-infected mice. In addition, NPNS treatment was associated with the downregulation of S-phase kinase-associated protein 2 (SKP2), a host factor known to facilitate intracellular viral replication. Collectively, these findings suggest that NPNS may serve as a first-line protective barrier during the early stage of influenza infection by simultaneously blocking viral entry and suppressing viral replication through its dual physicochemical and molecular mechanisms.

## 1. Introduction

The respiratory system is an essential organ for the vital activities of humans and animals. As it continuously channels air into the internal milieu, it is highly exposed to environmental pollutants and pathogens. Among various respiratory conditions, virus-induced respiratory diseases cause profound societal burdens and are primarily transmitted human to human via viral aerosols [1,2]. Upon infiltrating the nasal cavity, respiratory viruses rapidly disseminate through the nasal mucosa to the bronchi and lungs, rendering the entire respiratory tract highly susceptible to infection [3]. A prominent example is the influenza virus, which annually infects approximately 10% of adults and 30% of infants and young children [4,5]. Given that influenza variants, such as avian and swine flu, have historically threatened global public health, the strict control of this virus is an absolute necessity [6].

Consequently, extensive global research efforts have been dedicated to preventing and treating these viral respiratory infections. Conventional viral control strategies predominantly focus on vaccine-mediated prophylaxis, whereas post-infection therapeutic regimens heavily rely on small-molecule antiviral drugs, such as oseltamivir, nirmatrelvir/ritonavir, and molnupiravir [7,8]. However, the rapid emergence of unpredictable viral mutations continues to pose a severe threat to human health, underscoring the constant need for novel viral control strategies. In particular, as a rapidly mutating RNA virus, influenza presents significant clinical challenges due to its enhanced transmissibility, immune evasion capabilities, and the development of antiviral resistance [9]. Therefore, personal hygiene and preventive measures are emphasized as the primary defense strategies for public health, and there is an urgent need to develop broad-spectrum therapeutics applicable during the early stages of infection.

Moreover, strict adherence to personal hygiene cannot entirely eliminate the risk of viral transmission. Airborne respiratory viruses are highly likely to establish their initial infection at the nasal mucosa. Following mucosal colonization, the virus infiltrates the broader respiratory tract, where extensive viral replication occurs. Consequently, our ongoing research has strategically targeted the nasal cavity—the primary entry portal for respiratory viruses—as a focal point for preventive and therapeutic interventions [10]. Even if viral infection of the nasal mucosa has already occurred, effectively suppressing viral replication or preventing secondary spread during the early infection phase could significantly reduce the incidence and severity of respiratory diseases. Alongside widely utilized preventive and therapeutic strategies, novel pharmacological interventions specifically tailored for the nasal mucosa are urgently required, as they hold the potential to yield more clinically meaningful improvements in respiratory health outcomes.

Previously, we screened candidate substances capable of preventing or suppressing respiratory viral infections when applied directly to the nasal mucosa [10]. Based on these preliminary findings, we successfully developed and validated a prophylactic formulation combining xanthan gum (XG), a naturally derived polysaccharide, with camostat mesylate (camostat), a synthetic serine protease inhibitor [11]. Building upon this groundwork, the present study focuses on developing a therapeutic formulation for early-stage infections by combining XG with niclosamide, an anthelmintic agent historically utilized for tapeworm infections.

XG is a representative natural macromolecular polysaccharide produced through microbial fermentation; specifically, it is a high-molecular-weight anionic heteropolysaccharide synthesized by Xanthomonas campestris during carbohydrate fermentation. Structurally analogous to cellulose, it features side chains containing mannose and glucuronic acid, forming a negatively charged polyanionic structure. This configuration confers exceptional water retention capacity and robust stability against fluctuations in electrolytes and pH [12]. Due to these physicochemical properties, XG is extensively utilized across the food, pharmaceutical, and cosmetic industries. In particular, its excellent biocompatibility, high viscosity-forming capability, and structural stability across a broad pH range (1–13) and at temperatures exceeding 90 °C make it highly valuable as a vehicle for advanced drug delivery systems. Furthermore, XG exhibits distinct shear-thinning behavior, where its viscosity decreases under applied mechanical stress, making it highly advantageous for the formulation of sprayable therapeutics. Supported by our previous findings and established nasal mucus rheology studies utilizing artificial nasal mucus models [13], XG provides a compelling foundation for developing a therapeutic nasal spray targeting early viral infections. In this formulation, XG is employed not only for its intrinsic ability to prevent viral infection but also as a potent muco-adhesive agent to enhance the mucosal retention of the co-administered antiviral compound.

Niclosamide, originally developed in 1958 as an anthelmintic agent, exerts its anti-parasitic effects by uncoupling oxidative phosphorylation in tapeworm mitochondria, leading to fatal energy depletion [14]. Listed as a safe medication on the World Health Organization’s Model List of Essential Medicines [15], niclosamide has recently garnered significant attention as a potent intracellular viral replication inhibitor and has been investigated as a broad-spectrum antiviral therapeutic against SARS-CoV-2, respiratory syncytial virus, influenza viruses [16,17,18] and various cancers [19]. Mechanistically, niclosamide restricts viral proliferation by inhibiting S-phase kinase-associated protein 2 (SKP2). Viruses typically upregulate SKP2 protein to evade host autophagic clearance [20]. Consequently, we utilized niclosamide as a core active pharmaceutical ingredient for our novel intranasal therapeutic, leveraging its profound molecular antiviral efficacy.

The objective of the intranasal therapeutic developed in this study is a direct-acting nasal spray formulation. To achieve this, we formulated the niclosamide–polysaccharide nasal spray (NPNS) by co-formulating XG—which establishes a physical mechanical barrier against viral attachment and ensures prolonged mucosal retention of the antiviral agent—with niclosamide, which efficiently halts viral replication via SKP2 pathway inhibition. Currently available prophylactic nasal sprays under investigation and commercialization mainly include iota-carrageenan-based sprays (e.g., Viruseptin^®^) [21] and povidone–iodine-based sprays (e.g., COFIXRX^TM^) [22]. In addition, studies are actively being conducted to develop novel formulations by combining various active ingredients [23]. These products have been used as prophylactic agents for intranasal disinfection based on reports demonstrating their ability to inhibit viral activity. In this context, we aimed to develop an intranasal therapeutic formulation capable of exerting a more direct antiviral effect in virus-infected conditions.

We conducted comprehensive in vitro and in vivo studies to evaluate the distinct antiviral activities of these two active components including XG and niclosamide. For cellular assessments, GLP-compliant cytotoxicity assays were performed using murine fibroblasts (L-929), and antiviral efficacy was evaluated in human nasal epithelial cells (HNEcs) cultured in an air–liquid interface (ALI) model to accurately recapitulate the respiratory mucosal microenvironment. In vivo animal experiments quantified the time-dependent intranasal retention of the administered formulation, followed by evaluations of clinical symptoms, viral load, and SKP2 protein expression in influenza-infected mice treated intranasally with the test substance. Ultimately, this study demonstrates that NPNS provides a novel, dual-action antiviral strategy—integrating physicochemical barrier functions with molecular biological inhibition—offering a highly viable therapeutic intervention for patients in the early stages of respiratory viral infection.

## 2. Results

### 2.1. Antiviral Efficacy of Non-Cytotoxic NPNS in Virus-Infected Cells

**Figure 1:**
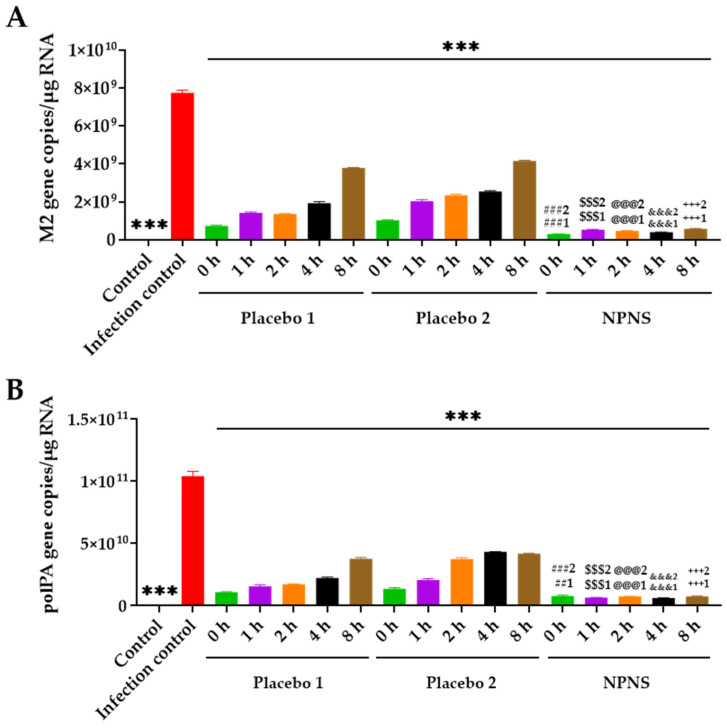
Quantitative evaluation of viral gene expression in influenza virus-infected HNEcs using an ALI model. All groups except the control group were infected with influenza virus, and the infected control group received no treatment. The other experimental groups were treated with placebo 1, placebo 2, or NPNS at 0, 1, 2, 4, or 8 h post infection. (**A**) *M2* gene and (**B**) *polPA* gene expression levels were evaluated at 2 DPI. Significance levels are indicated as follows: *** *p* < 0.001 compared to infection control, ^##1^ *p* < 0.01 compared to Placebo 1 at 0 h, ^###1^ *p* < 0.001 compared to Placebo 1 at 0 h, ^###2^ *p* < 0.001 compared to Placebo 2 at 0 h, ^$$$1^ *p* < 0.001 compared to Placebo 1 at 1 h, ^$$$2^ *p* < 0.001 compared to Placebo 2 at 1 h, ^@@@1^ *p* < 0.001 compared to Placebo 1 at 2 h, ^@@@2^ *p* < 0.001 compared to Placebo 2 at 2 h, ^&&&1^ *p* < 0.001 compared to Placebo 1 at 4 h, ^&&&2^ *p* < 0.001 compared to Placebo 2 at 4 h, ^+++1^ *p* < 0.001 compared to Placebo 1 at 8 h, and ^+++2^ *p* < 0.001 compared to Placebo 2 at 8 h. Data are presented as means ± s.d.; n = 3 per group.

The cytotoxicity of the NPNS formulation was initially evaluated. The negative control exhibited no detachment of intracellular granules, cell lysis, or growth inhibition, resulting in a reactivity grade of 0. Conversely, the positive control caused near-complete destruction of the cell monolayer, corresponding to a grade of 4. In the NPNS-treated group, cells displaying a loss of intracellular granules did not exceed 50%, and extensive cell lysis was not observed. Furthermore, cell growth inhibition remained below 50%, yielding a cytotoxicity reactivity grade of 2. According to ISO 10993-5 guidelines, materials with a reactivity grade of 0–2 are considered non-cytotoxic, indicating that NPNS does not induce cytotoxicity [24]. To evaluate the antiviral efficacy of NPNS under virus-infected conditions, we utilized an air–liquid interface (ALI) model designed to simulate the human nasal mucosal environment [25]. Following influenza virus infection of the established ALI model, the test formulations were administered at 0, 1, 2, 4, and 8 h post infection. The expression levels of the viral genes *M2* and *polPA* were analyzed at 2 DPI (Figure 1). For both the *M2* and *polPA* genes, the untreated infection control exhibited the highest expression levels, showing significant differences compared to all other experimental groups. All groups treated with either Placebo 1 or Placebo 2 demonstrated a reduction in *M2* gene (Figure 1A) and *polPA* gene (Figure 1B) expression relative to the infection control. Furthermore, a time-dependent decrease in the degree of viral gene suppression was observed as the time interval between infection and treatment increased. Although the difference was modest, Placebo 1 exhibited slightly lower viral gene expression compared to Placebo 2 when comparing groups treated at the same time points. Notably, the groups treated with NPNS at 0, 1, 2, 4, and 8 h post infection showed significant reductions in *M2* and *polPA* gene expression compared to the corresponding Placebo 1 and Placebo 2 groups at each respective time point. These results suggest that NPNS—a combination of XG and niclosamide—exerts a robust synergistic effect compared to the individual components, highlighting its potential to suppress initial viral infections more rapidly and potently.

### 2.2. Strong Intranasal Muco-Adhesion and Extended Residence Time of NPNS

**Figure 2:**
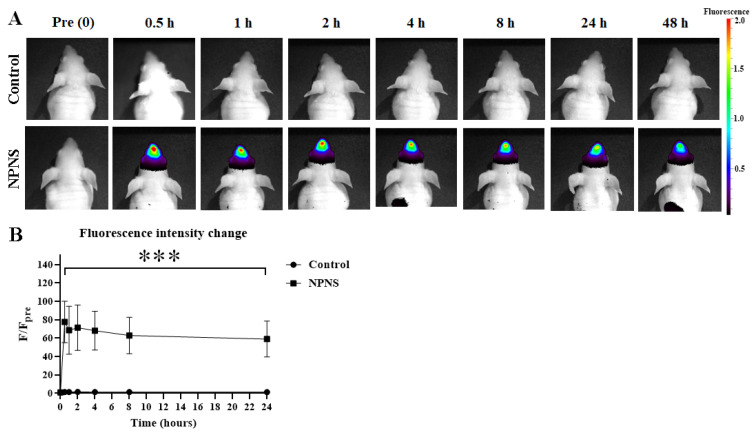
In vivo evaluation of intranasal muco-adhesion and extended residence time of NPNS. (**A**) Fluorescence images of the nasal region were acquired at 0.5, 1, 2, 4, 8, 24, and 48 h post administration using an in vivo imaging system (IVIS). Fluorescence intensity was quantified by selecting a standardized region of interest (ROI) within the nasal area and expressed as (p/sec/cm^2^/sr)/(µW/cm^2^). (**B**) In both the control and NPNS-treated groups, fluorescence intensities up to 24 h were normalized to their respective pre-administration (0 h) baseline values. Statistical significance is indicated as *** *p* < 0.001 compared to the corresponding control group (fluorescent dye solution only) at each respective time point. Data are presented as means ± SD; n = 5 per group.

To ascertain the practical applicability of NPNS in the nasal cavity, an in vivo animal study was conducted using mice. A non-viscous control solution containing only a fluorescent dye and the fluorescently labeled NPNS were administered into the nasal cavities of mice, and their film-forming and retention capabilities were evaluated from 0 to 48 h post administration (Figure 2). In the control group, the intranasal fluorescence signal diminished rapidly and became undetectable during the observation period. Conversely, in the NPNS-treated group, over 80% of the initial fluorescence intensity was maintained up to 8 h, and more than 75% of the signal was still detectable at 24 h post administration. These results demonstrate that the physical properties of NPNS are highly advantageous for mucosal adhesion and broad distribution within the nasal cavity, effectively ensuring the prolonged residence of the active antiviral pharmaceutical ingredients on the mucosal surface.

### 2.3. Therapeutic Efficacy of NPNS on Clinical and Pathological Symptoms in Influenza-Infected Mice

**Figure 3:**
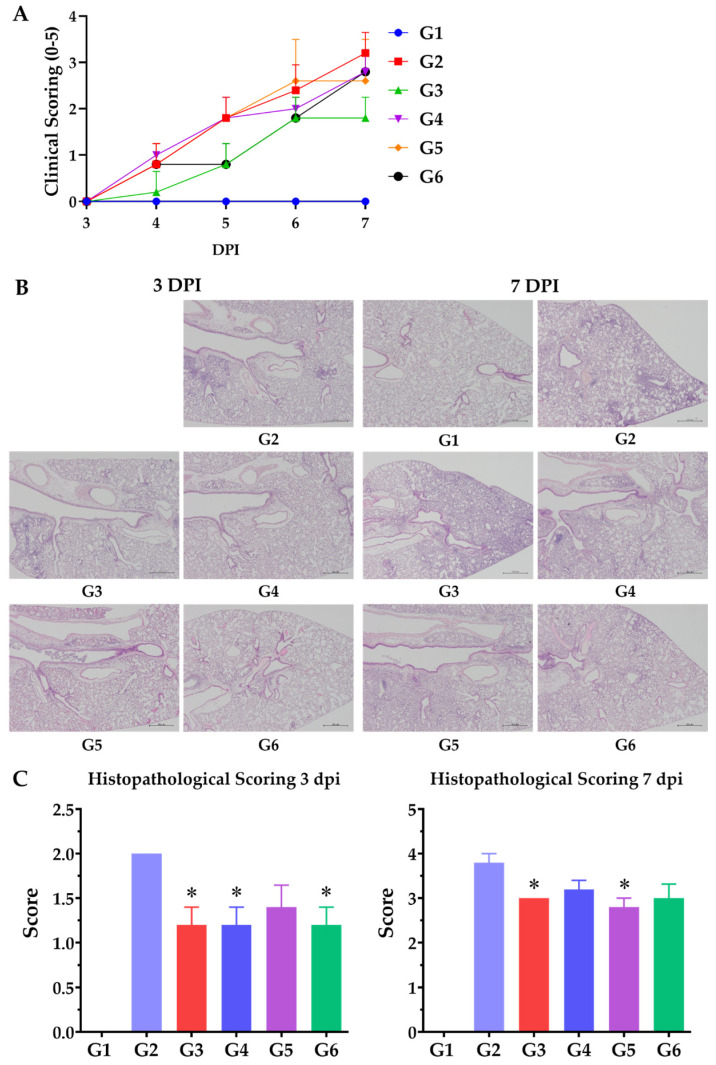
Clinical scoring and histopathological analysis of influenza virus-infected mice. Experimental groups were assigned as follows: G1, uninfected normal control; G2, untreated infection control; G3, oseltamivir-treated positive control; G4, G5, and G6, NPNS-treated groups at 1, 2, and 3 h post-infection, respectively. (**A**) Clinical scores recorded daily from 3 to 7 DPI following influenza virus infection. (**B**) Representative histopathological images of lung tissues (H&E staining) harvested at 3 and 7 DPI. Scale bar = 500 µm. (**C**) Pulmonary inflammation scoring based on the histopathological evaluation. * *p* < 0.05 compared to G2 (untreated infection control). Data are presented as means ± SD; n = 5 per group, except for G1 (n = 4).

To evaluate the therapeutic efficacy of NPNS against influenza infection in vivo, we established an animal infection model in which mice were intranasally administered NPNS at 1, 2, and 3 h post viral infection. Clinical changes were then monitored for up to 7 DPI (Figure 3). As depicted in Figure 3A, clinical symptoms began to manifest on day 4 post-infection. Overall, the positive control group (G3) treated with oseltamivir exhibited the mildest symptoms. Compared to the untreated infection control (G2), G3 showed improvements at 5 and 7 DPI. Furthermore, the G6 group, which received NPNS at 3 h post-infection, demonstrated improvement in clinical scores at 5 DPI compared to G2. Given that influenza-induced pulmonary inflammation is a major pathological outcome, histopathological analyses of lung tissues were conducted at 3 and 7 DPI (Figure 3B,C). The results indicated that all NPNS-treated groups exhibited reduced pulmonary inflammation compared to G2. Statistically significant reductions in lung inflammation were observed in G4 and G6 at 3 DPI, and in G5 at 7 DPI. These findings suggest that intranasal administration of NPNS, even post infection, can effectively ameliorate clinical symptoms and alleviate pathological pulmonary inflammation.

### 2.4. Suppression of Viral Replication by NPNS in Virus-Infected Mice

**Figure 4:**
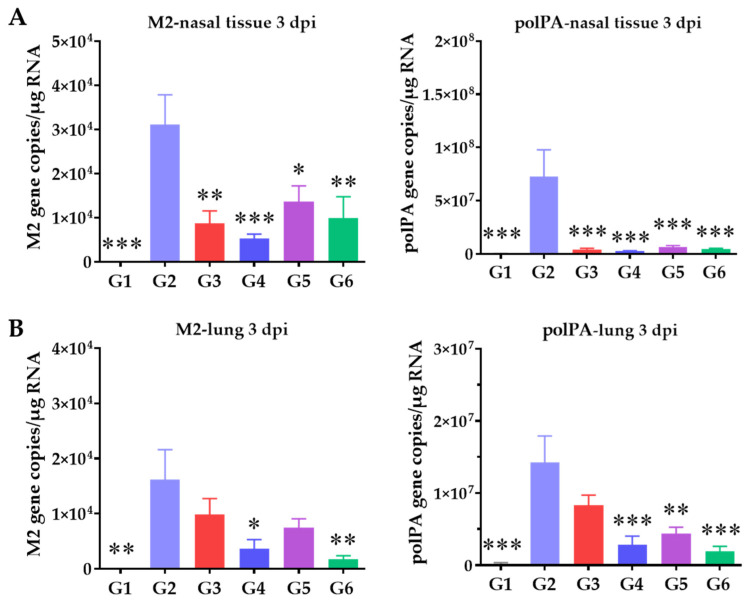
Quantitative evaluation of viral gene expression in the nasal and lung tissues of influenza virus-infected mice. Experimental groups were assigned as follows: G1, uninfected normal control; G2, untreated infection control; G3, oseltamivir-treated positive control; G4, G5, and G6, NPNS-treated groups at 1, 2, and 3 h post-infection, respectively. Expression levels of the *M2* and *polPA* genes were analyzed in (**A**) nasal tissues and (**B**) lung tissues at 3 DPI. Statistical significance is indicated as follows: * *p* < 0.05, ** *p* < 0.01, and *** *p* < 0.001 compared to G2 (untreated infection control). Data are presented as means ± SD; n = 5 per group, except for G1 (n = 4).

The expression levels of influenza viral genes in both nasal and lung tissues were analyzed during the acute infection phase at 3 DPI (Figure 4). In the nasal tissues, all treated groups exhibited significantly lower expression levels of both *M2* and *polPA* genes compared to G2 (Figure 4A). This indicates that the replication of the virus within the nasal tissues post infection was effectively suppressed by NPNS. In the lung tissues, the positive control group (G3) treated with oseltamivir did not show a statistically significant reduction in either *M2* or *polPA* gene expression. However, all NPNS-treated experimental groups demonstrated a significant decrease in both viral genes compared to G2 (Figure 4B). Collectively, the NPNS-treated groups exhibited significant reductions in viral genes in both nasal and lung tissues compared to G2, with a particularly distinct superiority over oseltamivir in the lung tissues. These results demonstrate that NPNS acts effectively in the nasal tissue—the primary site of viral replication—during the early phase of influenza infection (3 DPI) to suppress initial viral proliferation. Consequently, this potent local inhibition translates into a more definitive suppression of viral spread to the lung tissues, thereby preventing pulmonary damage, promoting recovery, and improving the overall disease course.

### 2.5. Antiviral Mechanism of NPNS via SKP2 Inhibition

**Figure 5:**
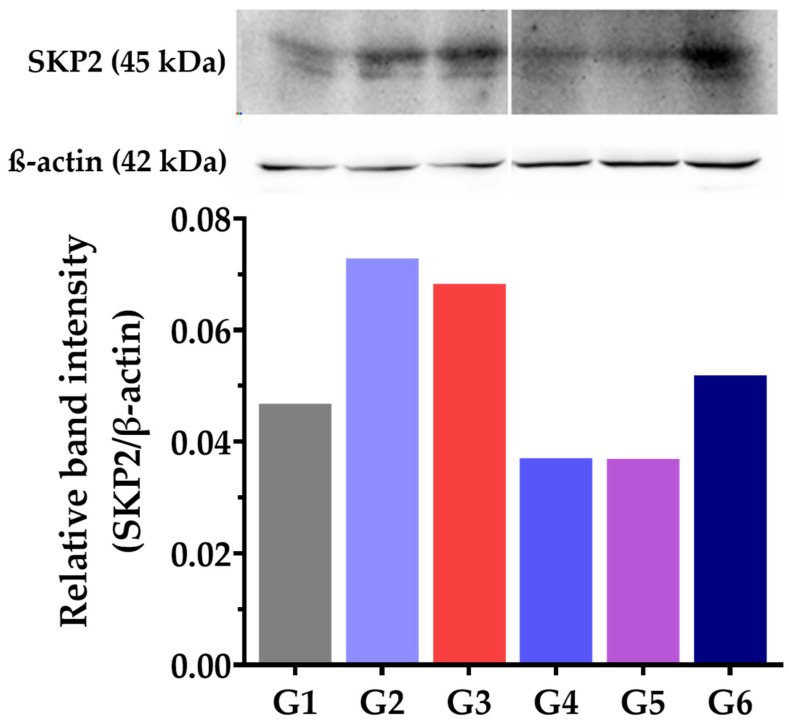
Western blot analysis of SKP2 protein expression in the nasal tissues of influenza virus-infected mice. Experimental groups were assigned as follows: G1, uninfected normal control; G2, untreated infection control; G3, oseltamivir-treated positive control; G4, G5, and G6, NPNS-treated groups at 1, 2, and 3 h post infection, respectively. Nasal tissues collected at 7 DPI were pooled by group to generate representative composite samples prior to protein extraction. SKP2 and β-actin bands were detected, and relative band intensities were quantified and normalized using ImageJ software.

To elucidate the antiviral mechanism underlying the inhibitory effects of NPNS, the protein expression level of SKP2 was analyzed. Nasal tissues collected at 7 DPI were pooled by group to prepare composite samples for analysis. The expression levels of SKP2, and β-actin were determined via Western blotting, and the results were normalized to the β-actin expression within the corresponding samples (Figure 5). The SKP2 expression levels in all NPNS-treated groups were reduced compared to both the untreated infection control (G2) and the oseltamivir-treated positive control (G3). G3 exhibited high SKP2 expression levels similar to G2, both being elevated relative to the uninfected control (G1). The most profound reductions were observed in the G4 and G5 groups, where SKP2 expression decreased by approximately 50% compared to G2 and by approximately 45% compared to G3, dropping to levels lower than those of the uninfected G1 group. In the G6 group, SKP2 expression was reduced by approximately 29% compared to G2 and by approximately 24% compared to G3. These findings strongly suggest that NPNS exerts its antiviral efficacy through the molecular inhibition of the SKP2 pathway, and that this inhibition is more effective when the formulation is administered closer to the time of initial infection.

## 3. Discussion

As a primary strategy for controlling most infectious diseases, including respiratory viral infections, prevention is often more effective than treatment. In densely populated urban environments, infection prevention through social distancing has proven effective in interrupting pathogen transmission [26], and blocking pathogens through enhanced personal hygiene has also been widely employed as a successful preventive strategy [27]. Accordingly, our previous research focused on developing prophylactic formulations aimed at preventing infection [11]. Nevertheless, when preventive measures fail and pathogens infiltrate the body, the secondary strategy relies on vaccination. However, the success of this approach is contingent upon the successful formulation of specific antibodies within the host, and it is limited by the unavailability of vaccines or the emergence of viral mutations that evade vaccine-targeted immunity. If vaccines fail to control the invading pathogens, the tertiary strategy involves therapeutic intervention, such as the prescription of conventional antiviral agents like oseltamivir [7]. Unfortunately, in cases of infection by viruses resistant to existing antiviral drugs, recovery depends entirely on symptomatic therapy relying on the individual’s immune system. Therefore, this study was conducted to propose a novel therapeutic strategy that can be readily utilized in daily life or administered concurrently with existing antiviral treatments.

Respiratory viruses predominantly enter the body through the nasal mucosa, which plays a critical role in the initial replication and transmission of the virus. Consequently, we have continuously investigated various influenza control strategies targeting the nasal mucosa [10,11]. Although prophylactic formulations for daily use have clinical value, their utility may become limited once infection has already occurred, as their effectiveness is likely to decrease under infected conditions. A study published in 2025 on acute respiratory infections demonstrated, based on both clinical and mechanistic evidence, that the timing of early intervention plays a critical role in improving patient prognosis and reducing socioeconomic burden [28]. Following our previous studies, which confirmed the feasibility of nasal sprays and screened prophylactic candidates, the present study aimed to develop a post-infection therapeutic agent applicable directly to the nasal mucosa. The fundamental formulation of NPNS was developed based on our previously studied camostat-containing prophylactic formulation. In accordance with the FDA guidelines for nasal spray excipients and formulation methods [29], niclosamide was incorporated as the active pharmaceutical ingredient. Because niclosamide is a poorly water-soluble drug, Hydroxypropyl betadex, a cyclodextrin-based solubilizing agent, was incorporated into the development of the NPNS formulation. During the formulation process, we visually confirmed that the inclusion of Hydroxypropyl betadex resulted in the dissolution or homogeneous dispersion of niclosamide within the formulation. This observation provides formulation-based evidence that Hydroxypropyl betadex increased the apparent solubility of niclosamide, thereby facilitating its incorporation into the formulation. However, since no analytical studies were performed in the present work to directly demonstrate the formation of an inclusion complex between niclosamide and Hydroxypropyl betadex, we cannot conclusively determine the exact physical state of niclosamide in the formulation. Therefore, we believe that Hydroxypropyl betadex contributes to the solubilization of niclosamide and that both molecular dissolution and cyclodextrin-mediated inclusion may contribute to the physicochemical state of niclosamide within the formulation. Initially, we established an ALI model using HNEcs to simulate the human nasal mucosal microenvironment. Following influenza virus infection in this model, the antiviral efficacy of NPNS was evaluated at both the transcriptional and translational levels. As shown in Figure 1, early post-infection treatment with Placebo 1 (containing XG as the sole active ingredient) or Placebo 2 (containing niclosamide as the sole active ingredient) resulted in a reduction in *M2* and *polPA* gene expression compared to the untreated infection control. However, as hypothesized, NPNS demonstrated a significantly more potent viral gene suppression effect than both placebos, driven by the synergistic interplay between the physicochemical barrier function of the polysaccharide XG and the molecular biological inhibitory action of niclosamide. We prepared and evaluated Placebo 1 and Placebo 2 to investigate the respective antiviral contributions of niclosamide and XG within the formulation. In particular, because Placebo 2 was prepared by removing only XG, it may influence not only antiviral efficacy and mucosal retention but also the physicochemical properties of the formulation, especially viscosity and spray behavior. However, physicochemical characterization of Placebo 2 was not comprehensively performed beyond the evaluation of pH and osmolality. Therefore, the direct impact of XG on the physicochemical properties of the formulation cannot be quantitatively assessed in the present study. We only confirmed that Placebo 2 did not exhibit significant differences in pH or osmolality compared with NPNS. Accordingly, we speculate that the major physicochemical differences between NPNS and Placebo 2 are more likely attributable to the inherent viscosity-enhancing and polymer matrix-forming properties of XG rather than to differences in acidity or osmolality. Nevertheless, this interpretation should be considered a plausible hypothesis rather than a definitive conclusion due to the lack of comparative physicochemical data. Placebo 2 was designed as an XG-free control formulation to evaluate the role of XG and was not intended to serve as a placebo formulation with physicochemical properties and spray characteristics identical to those of NPNS. Rather, it was used as a control formulation to assess the contribution of XG to the observed antiviral effects. For nasal spray formulations administered directly to the nasal mucosa, muco-adhesiveness is a critical factor [30]. To verify the practical applicability of XG and niclosamide in the nasal cavity, we synthesized fluorescently labeled NPNS and conducted in vivo animal experiments. A single intranasal administration of NPNS exhibited excellent mucosal adhesion and prolonged retention for up to 48 h (Figure 2). Based on the potent in vitro antiviral efficacy and in vivo muco-adhesiveness results, we proceeded with clinicopathological analyses in an in vivo influenza infection model (Figure 3). Over the 7 DPI observation period, the group treated with oseltamivir exhibited the most favorable clinical symptoms. Notably, the G6 group (administered NPNS at 3 h post infection) demonstrated a reduction in clinical symptoms at 5 DPI compared to the untreated infection control. Although clinical scoring in mice may be confounded by the discomfort associated with intranasal administration—potentially masking some expected symptomatic improvements—the histopathological results clearly demonstrated an alleviation of pulmonary inflammation comparable to that achieved by oral oseltamivir administration (Figure 3B,C). The superiority of the antiviral capability of NPNS was further confirmed in vivo through the quantification of viral gene expression in both nasal and lung tissues (Figure 4). At 3 DPI, NPNS treatment resulted in a significant reduction in viral gene expression in both nasal and lung tissues compared to the untreated infection control (G2). In contrast, oseltamivir failed to demonstrate a statistically significant reduction in viral gene expression in the lung tissues compared to G2 (Figure 4B). The FluV/A/PR8/34/H1N1 strain is widely used as a backbone strain for vaccine production and antiviral efficacy screening and is generally known to be susceptible to oseltamivir. Nevertheless, as shown in Figure 4B, oseltamivir did not significantly reduce viral RNA expression levels in lung tissues compared with the untreated infection control group. This finding may indicate that, during the early stage of infection, direct intranasal administration of NPNS more effectively suppresses viral propagation into the lungs than orally administered oseltamivir. Therefore, it is evident that the physical application of NPNS to the nasal mucosa exerts a synergistic effect—combining the mechanical viral entry blockade by XG with the molecular biological inhibition by niclosamide—thereby significantly reducing the viral load capable of infiltrating the lung tissues. To elucidate the underlying molecular mechanisms, we harvested nasal tissues at 7 DPI—a time point representing a mature infection and robust protein expression—and evaluated the expression levels of the SKP2 protein (Figure 5). SKP2 is an E3 ubiquitin ligase that regulates ubiquitin-mediated proteasomal degradation. It degrades Beclin 1, thereby inhibiting autophagy and preventing the host from clearing invading viruses [31]. The influenza virus notoriously induces autophagy during the early stages of infection to facilitate viral replication and assembly, but subsequently blocks autophagy in the later stages to maintain the infection [32,33]. Niclosamide has been identified as a promising broad-spectrum antiviral agent due to its ability to inhibit SKP2, thereby inducing and enhancing autophagy [18,34]. As depicted in Figure 5, the untreated infection control (G2) exhibited elevated levels of SKP2 compared to the uninfected normal control (G1). The oseltamivir-treated positive control (G3) also showed elevated expression levels of SKP2, with no significant difference from G2. Conversely, the NPNS-treated experimental groups demonstrated overall lower expression levels of SKP2 compared to G2 and G3. These results indicate that NPNS exerts its antiviral effects in influenza-infected mice by suppressing SKP2 expression to enhance viral clearance.

Taken together, these findings suggest that NPNS effectively suppresses early viral replication and blocks pulmonary infiltration through the molecular inhibition of the SKP2 pathway. Notably, viral clearance achieved by enhancing autophagy via SKP2 inhibition does not target a specific virus directly, which inherently lowers the risk of developing viral resistance and broadens its applicability as an early-stage therapeutic against a diverse range of respiratory viruses.

## 4. Materials and Methods

### 4.1. Preparation of NPNS Formulation and Placebos

The NPNS formulation evaluated in this study was prepared using the following protocol. Initially, potassium dihydrogen phosphate (0.68%; Merck, Frankfurt, Germany), sodium hydroxide (0.19%; Kirsch Pharma, Erzwaesche, Germany), and hydroxypropyl betadex (1%; Ashland, Industrieweg, The Netherlands) were dissolved in purified water and heated to 70 °C. Niclosamide (0.00001%; Derivatives Chemicals, Alcantarilla, Spain) was subsequently added and fully dissolved at 70 °C. Following this, L-menthol (0.02%; Anhui Fengle Perfume, Hefei, China) and benzethonium chloride (0.02%; Dishman Carbogen Amcis, Gujarat, India) were incorporated into the solution while gradually cooling it to 50 °C. At 50 °C, a 70% D-sorbitol solution (3.5%; Roquette, Lestrem, France) was added, and the mixture was further cooled to 25 °C. Once the temperature dropped below 30 °C, sodium hyaluronate (0.02%; Bloomage Biotechnology, Jinan, China) and xanthan gum (XG, 0.025%; Jungbunzlauer, Vienna, Austria) were introduced and completely dissolved. Each excipient in the present formulation was selected based on comprehensive considerations, including niclosamide solubilization, intranasal retention, spray suitability, preservative function, and nasal tolerability. Hydroxypropyl betadex was used as a solubilizing agent to increase the apparent solubility of niclosamide, a poorly water-soluble drug. Because niclosamide exhibits extremely low aqueous solubility, sufficient dissolution could not be achieved through simple heating and pH adjustment alone. Therefore, hydroxypropyl betadex was included to obtain a uniform dissolved state within the formulation. Benzethonium chloride was used as a preservative to suppress microbial contamination in the multi-dose liquid nasal formulation. Benzethonium chloride is a quaternary ammonium cationic preservative with antimicrobial activity against bacteria and certain fungi. In the present formulation, it was included not as an antiviral active ingredient, but rather to maintain the microbiological quality of the formulation. Menthol was incorporated to provide a cooling sensation and improve user acceptability during intranasal administration. However, because menthol may induce irritation depending on the concentration, it was used within a low concentration range intended to improve sensory acceptability while minimizing the potential for irritation. Sorbitol was used for osmolality adjustment and moisture-retaining support. Because the nasal mucosa may be sensitive to osmotic changes, sorbitol was included to adjust the osmolality of the formulation closer to the physiological range, thereby contributing to nasal mucosal tolerability. Sodium hyaluronate was used to enhance moisturization and mucosal compatibility of the nasal mucosa. Since the nasal mucosa is sensitive to dryness and irritation, sodium hyaluronate was intended to support moisture retention on the mucosal surface and provide a comfortable sensation after administration. Xanthan gum was used as the key muco-adhesive polymer in the formulation. Xanthan gum increases the viscosity of the formulation, prolongs intranasal residence time, and forms a protective adhesive layer on the mucosal surface, thereby allowing the drug to remain in contact with the nasal mucosa for an extended period. However, because excessive viscosity may reduce sprayability and user acceptability, xanthan gum was used within a range that maintained both muco-adhesiveness and sprayability. Potassium dihydrogen phosphate and sodium hydroxide were used as pH-adjusting agents, and their amounts were modified or additionally titrated to ensure that the final pH of NPNS was adjusted to approximately 7.5. From the perspective of nasal tolerability, the formulation was designed not by relying on any single excipient, but by comprehensively optimizing the final pH, osmolality, viscosity, moisturizing properties, and the concentrations of preservative and menthol to minimize the potential for mucosal irritation. The resulting homogeneous mixture was finally filtered through a 5 μm membrane filter as a clarification/polishing step to remove extraneous particulate matter that may have been introduced from raw materials, equipment, or the manufacturing environment during the preparation process, thereby yielding the final NPNS formulation. The physicochemical characterization of NPNS was conducted by CGBIO Co., Ltd. (Seongnam, Republic of Korea), and the formulation remained within the predefined acceptance specifications even after storage for 6 months under 50 °C/50% environmental conditions (Table 1). For comparative analyses, Placebo 1 and Placebo 2 were utilized; Placebo 1 was prepared using the identical protocol but omitting niclosamide, whereas Placebo 2 was prepared by excluding only XG. All materials used in these formulations were provided by the Research Center of Daewoong Pharmaceutical (Yongin, Republic of Korea).

### 4.2. Viruses

The influenza A virus strain (FluV/A/PR8/34/H1N1) was utilized for all in vitro and in vivo experiments. The virus was propagated by inoculation into the allantoic cavity of 9-day-old embryonated chicken eggs following standard procedures [35]. After incubation in the 9–10-day-old embryonated eggs, the allantoic fluid was harvested and stored at −70 °C. Viral titers were determined 48 h post inoculation and calculated as plaque-forming units per milliliter (PFU/mL), as described in a previous study [36]. For the in vivo studies, the virus was diluted to a concentration corresponding to 1 lethal dose 50 (LD50), equivalent to 150 PFU/mL, and administered intranasally (30 μL total volume) into the left nostril of the mice.

### 4.3. Cytotoxicity Assay

Cytotoxicity evaluations were conducted in compliance with ISO 10993-5 (Biological Evaluation of Medical Devices, Part 5: Tests for in vitro Cytotoxicity) and ISO 10993-12 (Biological Evaluation of Medical Devices, Part 12: Sample Preparation and Reference Materials) at the Korea Testing Certification Institute (Cheongju, Republic of Korea). Briefly, the test extracts were prepared by incubating 0.2 g of NPNS in 1 mL of Minimum Essential Medium (MEM; Gibco, Grand Island, NY, USA) supplemented with 10% fetal bovine serum (FBS; Gibco) at 37 °C for 24 h in a 5% CO_2_ incubator under constant agitation. A high-density polyethylene film (Hatano RI, Hadano, Japan) and 0.25% ZDBC polyurethane film (Hatano RI) were used as negative and positive controls, respectively, and were eluted under identical conditions at a ratio of 0.1 g per 1 mL of MEM. NCTC Clone 929 (L-929) murine fibroblasts, obtained from the American Type Culture Collection (ATCC, Manassas, VA, USA), were used for the assay. The experiment was initiated when the monolayer cultures in 6-well plates reached over 80% confluence. After aspirating the culture medium, 2 mL of each prepared extract was added to the wells, followed by incubation at 37 °C in a 5% CO_2_ atmosphere for 48 h. Cytotoxicity was assessed microscopically by evaluating cell morphology, vacuolization, detachment, cell lysis, and membrane integrity. The cytotoxic effects were graded on a scale of 0 to 4, with a grade of 2 or lower considered non-cytotoxic: 0, normal cell morphology; 1, slight (<20%) cell rounding, occasional lysed cells, or slight loss of intracellular granules; 2, mild (<50%) cell rounding or altered morphology without extensive cell lysis; 3, moderate (<70%) cell rounding and lysis with >50% growth inhibition; 4, severe, nearly complete or total destruction of the cell monolayer.

### 4.4. Virus Infection in HNEcs Under ALI Conditions

Human nasal epithelial cells (HNEc) were purchased from PromoCell (Heidelberg, Germany) and cultured at 37 °C in a 5% CO_2_ incubator using PneumaCult™-Ex Plus Medium (STEMCELL Technologies, Vancouver, BC, Canada). For seeding, 1 mL of the medium was added to the basal chamber of a 12-well Transwell^®^ insert plate (Corning, Kennebunk, ME, USA), and cells were seeded into the apical chamber at a density of 1 × 10^5^ cells/cm^2^. The plates were incubated until the HNEc monolayer reached confluence (approximately 2–3 days) [37]. To establish air–liquid interface (ALI) conditions, the apical medium was removed, and the basal medium was replaced with 1 mL of PneumaCult™-ALI Maintenance Medium (STEMCELL Technologies). The basal medium was refreshed every 48 h, and the cells were maintained under ALI conditions for 21 days prior to viral infection. For the antiviral efficacy assay, HNEcs were infected with the influenza virus at a multiplicity of infection (MOI) of 0.001. Subsequently, 140 μL of Placebo 1, Placebo 2, or NPNS was applied to the apical surface at 0, 1, 2, 4, or 8 h post-infection. To quantify viral gene expression (*M2* and *polPA*), total RNA was extracted at 2 days post infection (DPI) using Wizol™ Reagent (Wizbiosolutions, Seongnam, Republic of Korea), and analysis was performed as detailed in Section 4.9.

### 4.5. Mouse Preparation and Handling

Ten seven-week-old female Balb/c nude mice were obtained from Orient Bio (Seongnam, Republic of Korea) for the intranasal film formation assay. Additionally, 54 six-week-old female C57BL/6 mice were purchased from Samtaco, Inc. (Osan, Republic of Korea) for the in vivo influenza infection study. The animals were housed in filter-top microisolator cages under strictly controlled environmental conditions: 23 °C, 55% relative humidity, and a 12 h light/dark cycle, with ad libitum access to standard chow and water. A 7-day acclimatization period was provided, during which general health, feeding behavior, and growth were closely monitored. All animal procedures were approved by the Institutional Animal Care and Use Committees (IACUC) of the Asan Institute for Life Sciences and HLB bioStep, and were conducted in strict accordance with Institutional Biosafety Committee regulations and ARRIVE guidelines (Approval codes: Asan Institute for Life Sciences IACUC 2025-40-094; HLB bioStep IACUC 24-HB-0840).

### 4.6. Intranasal Film Formation and Retention Assay in Balb/c Nude Mice

Balb/c nude mice were anesthetized via inhaled isoflurane (Piramal Critical Care, Bethlehem, PA, USA). Prior to formulation administration, baseline background images were captured using an in vivo imaging system (IVIS; PerkinElmer, Waltham, MA, USA). To enable fluorescence tracking, NPNS was conjugated with Cyanine 5.5 amine (Lumiprobe, Cockeysville, MD, USA) in distilled water at a 10:1 volume ratio. The mixture was incubated overnight at room temperature to ensure complete conjugation. Subsequently, the reaction mixture was transferred to an Amicon^®^ ultrafiltration tube and centrifuged at 3000× *g* for 15 min at 4 °C. The filtrate containing unconjugated dye was discarded. The retained fluorescently labeled NPNS fraction was collected and dialyzed against distilled water at 4 °C for 6 h, with buffer exchanges every hour, to completely remove any residual free dye. Fluorescence quantification of the final dialyzed product confirmed a 0.4% binding efficiency of the dye. A control solution was prepared by dissolving Cyanine 5.5 amine in distilled water to match the 0.4% dye concentration. The fluorescently labeled NPNS and the control dye solution were administered intranasally at a dose of 20 μL per mouse (0.25 mg/kg) exclusively into the left nostril (n = 5 mice per group). Fluorescence images were sequentially acquired at 0.5, 1, 2, 4, 8, 24, and 48 h post administration using the IVIS. The fluorescence intensity within the nasal cavity was quantified by analyzing a standardized region of interest (ROI) [38].

### 4.7. Animal Study and Histological Analysis in C57BL/6 Mice

For the therapeutic efficacy study, NPNS was administered intranasally using a specialized spray dispersion syringe, while oseltamivir (Cipla, Mumbai, India), serving as the positive control, was dissolved in distilled water and administered orally. NPNS (20 μL per mouse/dose) was administered post viral infection and maintained three times daily at 8 h intervals. Oseltamivir (100 μg in 20 μL per mouse) was initiated 1 h post-infection and administered twice daily at 12 h intervals. Group G1 (n = 4) served as the uninfected, untreated normal control. All other groups (n = 10 mice/group) were infected with the influenza virus. The experimental cohorts were assigned as follows: G2 (negative control, infected but untreated); G3 (positive control, infected and treated with oseltamivir); and G4, G5, and G6 (infected and treated with NPNS starting at 1, 2, and 3 h post-infection, respectively). The observation period extended to 7 DPI. Clinical symptoms were scored daily using the following criteria: 1, slight ruffling of fur; 2, ruffled fur with reduced mobility; 3, ruffled fur, reduced mobility, and rapid breathing; 4, ruffled fur, reduced mobility, huddled posture, and labored breathing indicative of severe pneumonia; 5, death. Mice exhibiting severe respiratory distress or >20% body weight loss were humanely euthanized. At 3 and 7 DPI, five mice from each infected group were euthanized for tissue sampling; G1 mice were euthanized at 7 DPI. Under inhalation anesthesia, exsanguination was performed via transection of the abdominal aorta and inferior vena cava. Nasal tissues and whole lungs were excised. Half of each tissue sample was immediately frozen at −70 °C for molecular analyses (qRT-PCR and Western blotting). The remaining tissues were fixed in 4% neutral buffered formalin, dehydrated through a graded ethanol series, embedded in paraffin, sectioned at 4 μm, and stained with hematoxylin and eosin (H&E; Thermo Fisher Scientific, Waltham, MA, USA) for histopathological evaluation. Lung inflammation was graded based on established criteria [39]: minimal (scattered inflammatory cells), mild (aggregated inflammatory cells in <1/3 of the parenchyma), moderate (aggregated cells in 1/3 to 2/3 of the parenchyma), and severe (aggregated cells in >2/3 of the parenchyma).

### 4.8. Western Blots

To evaluate SKP2 expression, murine nasal tissues harvested at 7 DPI were washed three times with phosphate-buffered saline (PBS). Tissue samples from each group were pooled to create representative composite samples prior to protein extraction. Samples were homogenized and lysed in RIPA buffer (GenDEPOT, Katy, TX, USA) supplemented with a protease and phosphatase inhibitor cocktail (GenDEPOT) for 1 h. The lysates were cleared by centrifugation at 15,000× *g* for 15 min, and protein concentrations were determined using a bicinchoninic acid (BCA) assay. Equal amounts of protein (denatured at 100 °C for 5 min) were resolved by 10% SDS-PAGE and transferred onto polyvinylidene difluoride (PVDF) membranes (Bio-Rad, Hercules, CA, USA). The membranes were blocked with 5% non-fat skim milk for 1 h at room temperature and incubated overnight at 4 °C with primary antibodies against SKP2 and β-actin (1:1000 dilution; Abcam, Cambridge, UK). Following three washes with PBS containing 0.1% Tween-20 (PBS-T), the membranes were incubated for 1 h at room temperature with horseradish peroxidase (HRP)-conjugated secondary antibodies (goat anti-mouse or anti-rabbit IgG, 1:5000 dilution; GenDEPOT). Protein bands were detected using SuperSignal West Dura Extended Duration Substrate (Thermo Fisher Scientific) and visualized with a WSE-6200 Luminograph II imaging system (ATTO, Tokyo, Japan). Densitometric quantification was performed using ImageJ software (version 7.12; NIH, Bethesda, MD, USA).

### 4.9. Real-Time RT-PCR

Total RNA was individually extracted from nasal and lung tissues harvested at 3 DPI following a previously described method [40]. Tissue homogenization and RNA isolation were performed using Wizol™ Reagent (Wizbiosolutions). Quantitative real-time reverse transcription PCR (qRT-PCR) was executed on a CFX96 Real-Time PCR Detection System (Bio-Rad). First-strand complementary DNA (cDNA) was synthesized using the High-Capacity cDNA Reverse Transcription Kit (Applied Biosystems, Foster City, CA, USA). The 20 μL PCR reaction mixture comprised 2 μL of template cDNA, 10 μL of 2× Premix Ex Taq, and 200 nM of target-specific primers and probes. The sequences were as follows: for the influenza *M2* gene, forward 5′-CTT CTA ACC GAG GTC GAA ACG TA-3′, reverse 5′-GGT GAC AGG ATT GGT CTT GTC TTT A-3′, and probe [FAM] 5′-TCA GGC CCC CTC AAA GCC GAG-3′ [BHQ1]; for the *polPA* gene, forward 5′-CGG TCC AAA TTC CTG CTG A-3′, reverse 5′-CAT TGG GTT CCT TCC ATC CA-3′, and probe [HEX] 5′-CCA AGT CAT GAA GGA GAG GGA ATA CCG CT-3′ [BHQ1]. The thermocycling protocol included an initial denaturation at 95 °C for 30 s, followed by 45 cycles of 95 °C for 5 s and 60 °C for 20 s. No-template controls were strictly included in all runs. Samples were analyzed in duplicate, and amplification specificity was verified via melting curve analysis. Absolute viral RNA copy numbers were calculated utilizing a standard curve generated from known concentrations of viral cDNA, and the results were expressed as viral RNA copies per microgram of total RNA. Data acquisition and analysis were performed using Bio-Rad CFX Manager software (version 2.1).

### 4.10. Statistical Analysis

Statistical analyses were performed using GraphPad Prism 6 software (GraphPad Software, San Diego, CA, USA). Depending on data distribution, parametric or non-parametric methods were employed to compare group means. Viral titer data were analyzed using parametric one-way analysis of variance (ANOVA) followed by Dunnett’s multiple comparison test. Histopathological scoring data were evaluated using non-parametric methods, specifically the Kruskal–Wallis rank-sum test and the Mann–Whitney U test. A *p*-value of <0.05 was considered statistically significant.

## 5. Conclusions

Because the initial infection of respiratory viruses occurs at the nasal mucosa, targeting this site serves as the most efficient first-line therapeutic strategy. In this study, we developed and evaluated an intranasal therapeutic formulation designed for immediate, daily use upon early infection. The NPNS formulation developed herein demonstrated strong mucosal adhesion and effectively suppressed both the pulmonary infiltration and replication of the initially infecting virus through the targeted inhibition of SKP2. Based on this dual-action mechanism—integrating a physicochemical barrier with molecular biological inhibition—NPNS exhibits substantial practical potential as a non-invasive, self-administrable, broad-spectrum therapeutic intervention for early-stage respiratory viral infections.

## Figures and Tables

**Table 1:** The physicochemical characterization of NPNS.

No.	Evaluation Parameter	Acceptance Criteria
1	Delivered Dose per Actuation	Mean spray weight: ≥0.08 g per actuation over 10 actuations Individual spray weight: No more than 2 actuations below 0.08 g per actuation
2	Osmolality Test	150–350 mOsmol/kg
3	Loss on Drying	≥80%
4	Microbial Limit Test	Total aerobic microbial count (TAMC): ≤10^2^ cfu/g Total yeast and mold count (TYMC): ≤10 cfu/g Specified microorganisms (*Escherichia coli*, *Salmonella* spp., *Pseudomonas aeruginosa*, *Staphylococcus aureus*): Not detected
5	Film Formation Test	A film is formed on the surface and remains maintained after 30 min
6	Viscosity	1–10 mPa·s

## Data Availability

The raw data supporting the conclusions of this article will be made available by the authors on request.

## Abbreviations

The following abbreviations are used in this manuscript:

XG	Xanthan gum
HNEc	Human nasal epithelial cell
SKP2	S-phase kinase-associated protein 2
NPNS	Niclosamide–Polysaccharide Dual-Action Nasal Spray
PFU	Plaque-forming units
LD50	Lethal dose 50
ROI	Region of interest
ZDBC	Zinc Dibutyldithiocarbamate
GLP	Good laboratory practice
